# Inspection Digital Literacy for School Improvement

**DOI:** 10.3390/ejihpe13040053

**Published:** 2023-03-29

**Authors:** María del Carmen Martínez-Serrano, Manuel Angel Romero-García, Inmaculada García-Martínez, Óscar Gavín-Chocano

**Affiliations:** 1 Department of Pedagogy, University of Jaén, 23071 Jaén, Spain; 2 Andalusian Educational Inspection Service, 18011 Granada, Spain; 3 Department of Didactics, School Organization, University of Granada, 52005 Granada, Spain

**Keywords:** educational inspection, attention to diversity, accountability, digital literacy, digital competence

## Abstract

Educational inspection, as an essential part of the current educational environment, supports its mission through more pragmatic and comprehensive processes, techniques, and models, which guarantee the right of students to quality education. The aim of the present study was to determine the causal effect of gender and age on the dimensions of the instrument in the inspector population. Specifically, 118 male and female inspectors from the Educational Inspection Service of Andalusia (Spain) participated, with an average age of 47.56 years (±5.70). In terms of gender, 30 were women (25.40%) and 88 were men (74.60%). An instrument was developed specifically for this study with the purpose of assessing the participants’ opinions of the extent to which their work contributes to educational improvement. The results evidenced the relationship between the dimensions of the instrument: attention to members of the educational community (AMEC), supervision of guidance and tutorial action (SGTA), attention and inclusion of diversity (AID), and technological resources (TR) (*p* < 0.01). Similarly, the multigroup model obtained good structural validity (χ^2^ = 68.180; RMSEA = 0.078; GFI = 0.923; CFI = 0.959; IFI = 0.967). In terms of gender, no significant differences were obtained, although the results were moderately superior among males compared to females. In relation to age, younger inspectors had better TR results, and older inspectors had better AMEC and SGTA results. The conclusions strengthen the importance of the Education Inspection Service in educational establishments, highlighting the need to supervise the processes of attention and inclusion for diversity. A great deal of resistance was observed, especially as there is a lack of training in information and communication technology (ICT).

## 1. Introduction

Nowadays, the social development of knowledge within society has produced substantial changes in different spheres—particularly, in the field of education. An increasingly diverse, dynamic, and complex education demands new competencies, making a renewed, qualified, innovative, and—above all—practical educational inspection necessary when it comes to supporting educational institutions [1].

Educational inspection is a “forgotten” field in social science research, despite being an instrument that most educational systems use to promote and evaluate the quality of education [2]. In this regard, most studies centered on accountability focus on teachers [3], management [4,5], or educational reforms designed by educational governments [6].

### 1.1. Educational Inspection and Accountability

Educational inspection has among its functions and competencies the development of different actions with the aim of effectively and practically guaranteeing compliance with the principles set out in the education laws of their corresponding countries [7]; among the most significant of these is to promote the right to education and ensure compliance with the duties and functions of the different educational agents in order to guarantee attention for the needs arising from the diversity of students, who are naturally heterogeneous [8,9,10]. In short, educational inspection should help schools by empowering teachers to take an active role and meet the proposed objectives [11] while establishing bridges with management so that they implement timely changes within the school institution they lead. However, a tendency is being observed to reduce inspection to educational bureaucratization. Adopting constructive and school improvement approaches implies a deep rethinking about where we are going, but it also calls for the systematization of evaluation processes. In this regard, there are studies that have examined the important role of certain actions (such as the use of “templates” in the evaluation of schools) where greater transparency in the analysis of educational reality and quality is demanded [12].

In the Spanish context, key parts of their attributions are the supervision of tutorial action and educational and vocational guidance, both of which are elements that favor the inclusion and permanence of students in different educational stages with guarantees of success [9]. In parallel, internationally, there is evidence of the need to promote adequate digital literacy that allows for the development of essential competencies for the integration of students, as citizens, into 21st-century society [13]. These the Spanish financial budgets for education are in line with the recommendations established by the European Union Council 2018/C 189/01 of 22 May 2018 regarding key competencies for lifelong learning; since more and more jobs have been automatized, and technologies have greater relevance in all areas of work and life.

Thus, educational inspectorate must have enough resources to actively participate in technical supervision tasks. In order to perform these tasks properly, it is necessary to strengthen aspects related to the technological use of information, as a new challenge, in order to quickly and effectively reach the whole educational context. This is achieved by promoting educational actions linked to the problems of schools, which allows for the proposal and development of training actions that involve the different educational agents, their organizations, and their associations [14].

In this regard, one element of the inspection quality will be measured through initial training for candidate inspectors and ongoing training for those in service [15,16]. These functions are carried out through different actions, since their objective is the collection of evidence and its subsequent analysis, in order to advise mainly managers and teachers. Much of this evidence is collected in platforms [1] and other resources included in the inspection plan of the Junta de Andalucía, Spain [17,18]. Hence, members of the inspection services should incorporate digital competence into their professional competencies in order to improve their actions [19].

In a similar vein, it has been observed that the inspection services from different European countries have a systematic impact on the the improvement of school performance at different levels; therefore, it has been detected that school principals (who are the subject of supervision actions included in the respective plans) feel more “pressure of responsibility”. As a result, they are more attentive to the quality expectations reported by the inspections, more sensitive to the reactions of stakeholders (teachers, families, students, etc.) and the results of the inspections, and more active with respect to improvement activities. However, the number of unintended consequences also increases with pressure. School leaders consider that inspection systems in different countries apply differential degrees of “accountability pressure”, which is reflected in the system-specific amounts of improvement activities [20].

In the European context, educational inspection, as happens in Spain, carries out the instrumental mission of examining, evaluating, and helping schools to comply with legislation to ensure the quality of education in all areas [21]. This is completed by providing the help and advice necessary to carry out the tasks, which serve to guarantee high-quality schooling [22].

From the development perspective of different educational reforms, studies have suggested the need to integrate the monitoring tasks of school inspections within accountability systems based on the evaluation of the system’s functioning by specialized agents and/or external evaluations [23]. Accountability has been increasingly emphasized as key to improving quality and inclusion in educational institutions [22]. Although most of the literature refers to the need for monitoring of all innovation processes, through different collegial bodies, there are also studies that point out that inspection action also causes certain negative effects. However, it is complex to analyse these adverse effects due to the important role of contextual factors in the field of education [24].

### 1.2. Educational Inspection and the Importance of Promoting Digital Skills

Today’s society is eminently digital. Indeed, in the labor market, so-called digital competencies have positioned themselves as most important in the path to achieving greater employability. Educational institutions, in response to these demands, have also begun to integrate digital competencies, in recent years, in the basic curricula and study programs at different educational stages, with the intention of providing students with the necessary tools to develop successfully in life.

In order to know the origin of these competencies, it is necessary to go back more than two decades. In an attempt to converge the necessary knowledge that 21st century citizens need for their success, digital competencies emerged in 2006 as part of the recommendations promoted by the European Parliament as Key Competencies for Lifelong Learning (OJ L394, 2006). Similarly, within the convergence of the European Higher Education Area, the Turnitin project arose, within which the importance of promoting a framework for digital training among teachers was demanded [25]. Thus, organizations, such as UNESCO (2011), published a framework of teaching competencies that was structured in three major blocks—namely, technological literacy, knowledge deepening, and knowledge creation.

From this moment onward, different studies and training frameworks have emerged with the perspective of digitally training teachers and educational institutions to be able to meet the demands of an emerging digital environment. An example of this is the study entitled “DIGCOMP: A Framework for Developing and Understanding Digital Competence in Europe” [26], which has the purpose of advancing the understanding and acquisition of digital competence. Bearing in mind the important role played by schools in the education and training of students, educational institutions are positioned as those spaces that must meet the necessary conditions to transmit and acquire such learning. Considering the importance of establishing a digital teacher model, different studies have joined efforts to develop a framework that brings together the digital qualities that all effective teachers must possess [27,28,29] worldwide. Likewise, different organizations, such as the National Institute of Educational Technologies and Teacher Training (INTEF), have based their studies on the recommendations derived from DIGCOMP, as well as from the main findings reported in the specialized literature. Among their contributions, the division of digital competence into five clearly delimited competency areas stands out [30]: information and information literacy, communication and collaboration, digital content creation, security, and problem-solving.

The European Framework for Teachers’ Digital Competence (hereafter DigCompEdu), details, in 6 areas, the 22 digital competencies; areas 2 to 5 are those that constitute the pedagogical core of the framework, which all educators at all educational levels should aspire to reach [31]. In this sense, [25] provides a rather positive assessment of such a framework, as it is flexible enough to adapt to different educational institutions, and, on the other hand, to understand the current digital competence levels of teachers and, thus, be able to improve them from their initial training. Thus, different experiences are being carried out to promote the digital literacy of every individual involved in the teaching–learning process [27] and different people working in educational institutions [29].

Although inspectors fall within the teaching staff, their tasks are different from these; therefore, their level of digital competence, as well as the ability to analyze large volumes of data [16] is very relevant to achieving improvements in the educational system.

Based on what has been previously reported, the present study aimed to achieve the following objectives: (a) analyze the existence of significant correlations between each of the dimensions of the evaluation instrument; (b) establish the existence of significant differences in the dimensions of the instrument considered and the sociodemographic variables of gender and age; (c) determine which dimensions of the instrument used (supervision of guidance and tutorial action, attention to members of the educational community, and technological resources) predict greater attention to and inclusion of diversity; and (d) study the causal effect of each of the dimensions described, according to gender, through a structural equation multigroup model (SEM).

## 2. Materials and Methods

This descriptive study is based on cross-sectional, non-experimental, and correlation quantitative analyses. Based on these criteria, measures of the longitudinality, comparativeness, and reliability of the scores were established through the calculation of Cronbach’s alpha and the omega coefficient, also known as Jöreskog’s Rho [32].

### 2.1. Participants

The study sample was composed of 118 inspectors belonging to the Educational Inspection Service of Andalusia (Spain). A causal, non-probabilistic, convenience sampling method was carried out. The only inclusion criterion used was that they should be active inspectors in the Andalusian community, so the participants were people who agreed to participate in the study voluntarily. The distribution of the sample by sex was 30 women (25.40%) and 88 men (74.60%), which is consistent with the trend reported by the National Institute of Statistics. In terms of age, the participants were between 32 and 63 years old, with a mean age of 47.56 years (±5.70).

### 2.2. Instrument

An instrument was developed specifically for this study, and its purpose was to assess the opinions of Andalusian education inspectors regarding how much their work contributes to school improvement [33]. The questionnaire consisted of two clearly differentiated parts. First, there was a sociodemographic questionnaire, in which potential participants were asked about their sex, age, and years of professional experience in different professional positions. This was followed by a Likert-type rating scale of 27 items and 5 response options (1 = not at all; 2 = a little; 3 = neutral; 4 = quite a lot; and 5 = a lot). The overall reliability of the scale was high (Cronbach’s alpha = 0.890). The factor analysis carried out reported 4 factors that explained 51.95% of the total variance.

Attention to the members of the educational community, which includes nine elements, refers to the interactions of different members of the educational community in order to supervise tutorial action and advise them about their rights and responsibilities in the educational process. It also involves supervising related documents and provides monitoring for attention to diversity.

Supervision of guidance and tutorial action groups together seven elements and relates to the support provided to teachers, who are responsible for tutorial action and educational and vocational guidance, as well as families.

Attention to and inclusion of diversity includes six elements and refers to the contributions made by the inspection service to improve school absenteeism, school drop-out rates, individualized attention, and the integration of families within school life, as well as the improvement of the information they receive.

Technological resources consist of five elements. This factor includes the inspectors’ assessment of the resources included in the plan, such as educational platforms, e-mail, smartphones, and videoconferencing.

### 2.3. Procedure

Participants were contacted through a letter sent to the person in charge of each Provincial Education Inspection Service via email or in person, depending on the location of the service. The letter explained the purpose of the study and included a request to collaborate on the study and its dissemination. The number of questionnaires sent to each service differed according to the number of inspectors assigned to them. All the provincial services returned completed questionnaires except for one.

Prior to completing the questionnaire, participants were informed about the purpose of the study and the instructions for survey completion, and their confidentiality and anonymity were assured in accordance with the ethical guidelines recommended by the Declaration of Helsinki [34]. The instrument was then provided in a sealed envelope for subsequent mailing.

### 2.4. Data Analysis

To achieve a better fit for each of the instruments, the data were transformed according to their factor loadings [35]. Next, descriptive data (means and standard deviations) were calculated to analyze, a priori, the reliability and internal consistency of each instrument through Cronbach’s alpha and the omega coefficient. We worked with the weighted sum of each variable by overcoming limitations that could affect the proportion of variance [36] and the correlation between the data obtained in each of the established variables. Parametric tests were used, as the assumption of normality was met in all cases based on the results obtained with the Kolmogorov–Smirnov test (n > 50 cases). In addition, the effect sizes for the analyses performed are reported. Data analyses were conducted following the parameters of the general research model for correlation and regression analyses [37]. Next, analyses of mean differences, according to gender and age, were performed using Student’s *t*-test and an ANOVA to calculate the mean differences for unrelated samples.

## 3. Results

The hypotheses of multicollinearity, homogeneity, and homoscedasticity were evaluated to determine whether the resulting distribution met the criteria for interdependence between variables. A confirmatory factor analysis (CFA) was carried out using data collected with the different instruments (see Table 1) in order to validate and examine the internal structure of each item.

Table 2 provides the scores from the correlation matrix (Pearson correlations were conducted since the distributions were normal), the descriptive statistics (mean and standard deviation), and the reliability analyses for the scores (Cronbach’s alpha and the omega coefficient), which generally show a good level of reliability for the scores obtained.

Upon analyzing each of the dimensions of the instrument used, a statistically significant relationship was observed between the dimensions; the highest correlation was established between the dimensions of attention to members of the educational community and attention to and inclusion of diversity (r(118) = 0.54; *p* < 0.01).

### 3.1. Differences According to the Sociodemographic Variables of Gender and Age

To analyze differences in relation to gender, Student’s *t*-test (mean differences) of independent samples was performed (see Table 3). The results indicated that there were no significant differences among any of the dimensions of the instrument used with respect to the gender variable (*t* < 2.0; *p* > 0.05, ns), with slightly higher scores for women than for men.

To analyze differences according to age, four intervals were determined (<41 years, 41–50 years, 51–60 years, and >60 years), and an ANOVA was performed (see Table 4). Significant differences were found among the dimensions of attention to members of the educational community [F(3.58), *p* = 0.011]; supervision of guidance and tutorial action [F(6.05), *p* = 0.015], and attention to and inclusion of diversity [F(5.26), *p* = 0.021]. The post hoc test performed (Tukey’s HSD) reported that there were statistically significant differences between some of the intervals; the effect sizes were small in all cases (*η*^2^) and (*ω*^2^). Specifically, younger inspectors had better TR results, and older inspectors had better AMEC and SGTA results.

### 3.2. Hierarchical Multiple Regression Study

In order to demonstrate and quantify the predictive capacity of the dimensions (attention to members of the educational community, supervision of guidance and tutorial action, and technological resources on attention to and inclusion of diversity (See Table 5), a hierarchical multiple regression analysis was performed. Discarding, a posteriori, those variables that did not fit the proposed regression model and verifying the absence of multicollinearity problems (the aceptable values were <0.20; IVF > 4.00 and 0.20; IVF > 4.00), our values ranged between 0.832 and 1.201. The results of the Durbin–Watson test indicated that there was an independence of errors, with a value of 2.125. Given that the value was between 1 and 3, we accepted the assumption.

The dimensions included in the regression model explained 41.0% of the variance, with the variable attention to members of the educational community serving as the best predictor of attention to and inclusion of diversity (R = 0.567; R^2^ corrected = 0.304; F = 27.251), with significant *t*-values for the rest of the variables.

### 3.3. Multi-Group or Multi-Sample Structural Equation Modeling

Model fit was verified using the chi-square (χ^2^) value, the goodness-of-fit index (GFI), and the root mean square error of approximation (RMSEA) as measures of absolute fit. The adjusted goodness-of-fit index (AGFI), the Tucker–Lewis index (TLI), and the comparative fit index (CFI) were used as measures of incremental fit. The chi-square ratio (χ^2^) over degrees of freedom (CMIN/GL) and Akaike’s information criterion (AIC) were used as measures of parsimony fit [38].

In the first instance, the validity and fit of the model established from the resulting data in the regression analysis were checked and presented a significant chi-square (χ^2^) value (χ^2^ = 68.180; gl = 46; *p* = 0.001). However, this statistic is sensitive to sample size and should be interpreted with caution. In this regard, different studies suggest the use of other estimators to evaluate model fit [39]. Among the most commonly used, we highlight the goodness-of-fit index (GFI), which had a value of 0.923, showing an acceptable model fit, as well as the value of the comparative fit index (CFI), which obtained a value of 0.959. The incremental fit index (IFI) obtained an acceptable value of 0.967. The adjusted goodness-of-fit index (AGFI) presented a value above 0.85, which also suggested a good fit. Finally, the root mean squared error (RMSEA) indicated an anticipated fit with the total population value, being lower than 0.08 for the established parameters. The values of this index were proposed by Steiger and Lind [40], who suggested compensating for the effect of model complexity by dividing by the number of degrees of freedom to test the model. Values below 0.08 are indicative of a good fit, and in our case, it was 0.078. Consequently, the model fit was acceptable in relation to the data obtained. Given the lack of balance in our sample with respect to sex, coinciding with the proportions found for the analysis sample, we chose to present two models.

**Figure 1:**
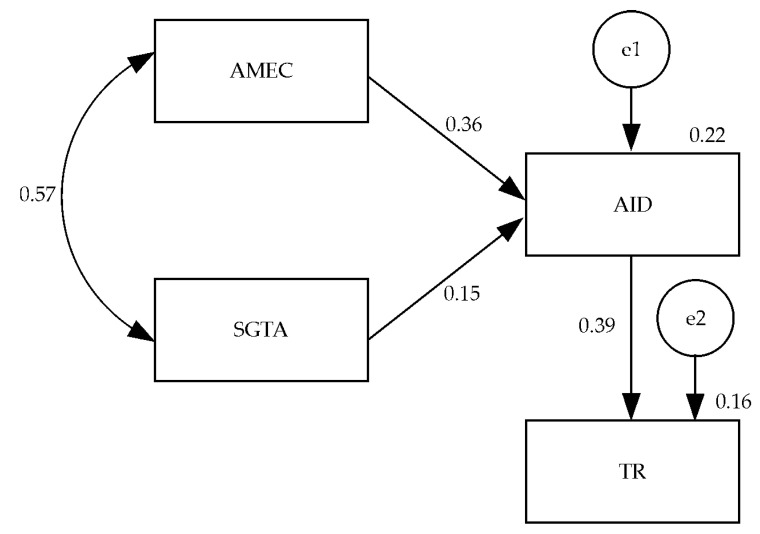
Multi-group structural equation model for men.

Figure 1 shows the standardized weights between each of the variables for men, where a significance level of 0.005 (5% probability of error) was established for the indicators with the highest regression weights of the variables being below this value. 

According to the Table 6, it found that attention to members of the educational community on attention to and inclusion of diversity (3.107) and attention and inclusion to diversity on technological resources (3.984). Further, a significant relationship was found between the dimensions “attention to members of the educational community” and “supervision of guidance and tutorial” action (4.629).

**Figure 2:**
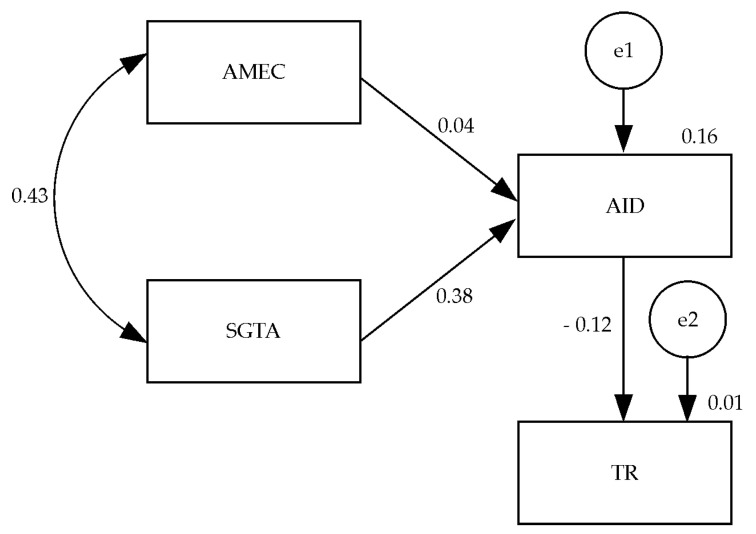
Multi-group structural equation model for women.

Figure 2 shows the standardized weights between each of the variables for women, with an established significance level of 0.005 (5% probability of error). The indicators with the highest regression weights for the variables were below this value (see Table 7); this corresponded to the influence of supervision of guidance and tutorial action on attention to and inclusion of diversity (2.023). Further, a significant influence was found between the dimensions attention to members of the educational community and supervision of guidance and tutorial action (2.149).

## 4. Discussion and Conclusions

The purpose of this study was to analyze the existence of significant correlations between each of the dimensions of the evaluation instrument and to determine which dimensions (attention to members of the educational community, supervision of guidance and tutorial action, and technological resources) predicted greater attention to and inclusion of diversity. Similarly, the aim was to examine whether there were statistically significant differences according to sex and age.

First, the reliability (internal consistency) of each of the instruments was verified through Cronbach’s alpha calculations. It is the most commonly used value; subsequently, the omega coefficient was used, as it is the most appropriate estimate when there is a disparity in the factorial loadings for each item (Tau equivalence) because it works the weighted sum of each variable and overcomes the limitations that could affect the proportion of variance [36,41].

In general, the results were consistent with other papers [9,10,41], even though they used different instruments. The relationship established among the attention to and supervision of members of the educational community with the level of competence acquired in the field of ICT is highlighted, with attention to and inclusion of diversity placed at lower levels. These results fully agreed with the findings of other studies, where it has been shown that educational inspection assumes a transforming role in the use and training of technological aspects, and they are perceived as less competent in effectively integrating the demands required for a diverse and inclusive school [2]. Specifically, the advisory role of the inspection toward different members of the educational community, as well as the supervision of tutorial action plans, favors the design of an educational response adjusted to the demands of today’s society. Furthermore, it favors the promotion of inclusive measures for all students, while promoting innovation in educational establishments through the use of technological resources.

The supervisory function is exercised by members of educational inspection services as an instrument to be used to raise the levels of educational quality and equity [42]. The inspector’s work consists of carrying out the planned, continuous, and comprehensive supervision of the procedures carried out in/by the educational institutions and classrooms by qualified professionals with authority in these matters [43]. During this supervision, the implementation of the axes that make up the inspection plan, which has the purpose of ensuring improvement in the educational quality of the system, is attended to [33].

The analyses carried out to determine whether there were correlations between the dimensions (objective 1) showed that there were, indeed, significant relationships between all of them (due, in part, to their networking methodology and their capacity for collaboration), especially between the dimensions of attention to members of the educational community and the attention to and inclusion of diversity. It was confirmed that the monitoring that was carried out in the attention to and inclusion of diversity dimension is a primary core that involves the assessment of all the actions carried out in relation to the improvement of and inclusion of students in the educational system [9].

Likewise, other studies show that the use of digital technology promises positive impacts on the social and economic integration of young adults with developmental disabilities, the support of their autonomy, and the facilitation of their inclusion in society [44].

In relation to our second objective, it was partially confirmed in relation to sociodemographic variables, since the results ratify significant differences according to age (among the dimensions of attention to members of the educational community, supervision of guidance and tutorial action, and attention to and inclusion of diversity); however, no significance was found for gender, although there is a better perception of these actions by women, whose scores were slightly higher [45]. This result was also found in another study, which provides empirical evidence that the age of inspectors is a variable that affects attention to diversity in secondary schools [9].

Regarding the third objective of determining which variables of the instrument used (attention to members of the educational community, supervision of guidance and tutorial action, and technological resources) predicted greater attention to and inclusion of diversity, a stepwise linear regression analysis was performed, discarding those dimensions with non-significant values. In our case, the EI variables that entered the model were: attention to members of the educational community and supervision of guidance and tutorial action. These data corroborate other findings suggesting that there is a connection between educational inspection and the institutional context of schools [20]. However, there is still a long way to go in terms of determining its role in the current model of 21st-century schools, including strategic activities in the use of ICT [2], attention to diversity [46], and the strengthening of an inclusive curriculum [20,47].

Finally, a multigroup analysis was performed with the multivariate statistical technique of structural equations, to check if there were significant differences in each of the variables, displaying a good model fit, which evidenced a good relationship between attention to members of the educational community and supervision of guidance and tutorial action. The mediating role of attention to and inclusion of diversity with the use of technological resources was also significant in terms of gender, where the result was moderately higher for men than for women. It is likely that the evidence is conditioned by the greater number of men in the sample, which would hinder the resulting validity. Similarly, these results could be contingent on the inspection of education’s perception of attention to diversity [2,20,48]. Therefore, the data should be taken with caution to avoid misinterpretation of the current inspection system in Spain.

Despite the important contribution of the study, it is important to point out that it also has several limitations that should be considered prior to the assumption and generalization of the findings obtained. Thus, the research design is positioned as the first, since it is a cross-sectional study, in which the instrument was administered only once. For this reason, it does not offer an understanding of reality or an identification of the causes that can be derived from the results obtained. Accordingly, and consistent with the specialized scientific literature on research designs, it would be relevant to develop longitudinal studies with this population, with the intention of exploring and learning about the impact that the actions and the context in which the inspectors operate have on their professional development and educational quality. The sample size can be considered another limitation, as it is relatively small. In this regard, further research should increase the number of participants in order to be able to generalize the results obtained. Likewise, this study only contains the inspectors’ perception of a concrete reality. However, in order to carry out a more reliable analysis of the impact of inspectors’ actions on educational improvement, future studies will also include the assessment of other members of the educational community, such as teachers, students, and families. Based on the findings obtained, it is necessary to identify a set of characteristics that should be taken into account in order to achieve greater inspection professionalism. Thus, the inspection should guarantee the responsible and safe use of ICT in schools, while ensuring the right to privacy and digital security. This is crucial in order to promote inclusive and sustainable digital practices in schools in line with the provisions of different frameworks, such as Digcompedu [25]. Consequently, the implementation of these actions can be encouraged and strengthened by evaluations carried out by the inspection regarding the impact of ICT on teaching practices and by the promotion of training based on ICT and innovation. In turn, the accessibility provided by ICT is another initiative that should be promoted by the inspectorate, based on clear policies and protocols that must be followed by the different members of the educational community.

## Figures and Tables

**Table 1:** Factor loadings.

Indicator	α	ω	Estimate	SE	Z	*p*	*β*
Attention to members of the educational community (AMEC)							
Supervision of the Tutorial Action and Guidance Plan (POAT) serves to improve tutorial action and educational guidance.	0.874	0.891	0.319	0.0387	8.26	<0.001	0.696
Supervision of the Transition Program serves to improve tutorial action and educational guidance.	0.870	0.887	0.374	0.0389	9.63	<0.001	0.778
Interviews and meetings with school management teams and department heads serve to improve tutorial action and educational guidance.	0.873	0.889	0.270	0.0328	8.22	<0.001	0.695
Interviews with educational guidance personnel serve to improve tutorial action and educational guidance.	0.875	0.892	0.337	0.0411	8.20	<0.001	0.692
Interviews with parents or their representatives serve to improve tutorial action and educational guidance.	0.877	0.893	0.178	0.0289	6.17	<0.001	0.553
The development, submission, and presentation of standardized reports improve tutorial and vocational guidance.	0.877	0.894	0.221	0.0424	5.20	<0.001	0.478
Monitoring of incidents serves to improve tutorial action and educational guidance.	0.876	0.893	0.291	0.0493	5.89	<0.001	0.532
Teacher counseling improves attention to diversity and educational guidance.	0.875	0.890	0.195	0.0290	6.74	<0.001	0.594
Supervision of guidance and tutorial action (SGTA)							
Inspection actions allow for improved family participation in school life.	0.875	0.892	0.185	0.0301	6.16	<0.001	0.584
Support for the tutorial function of teachers improves educational guidance.	0.874	0.891	0.189	0.0405	4.66	<0.001	0.472
Inspection actions have allowed for improved vocational and professional guidance for students.	0.875	0.892	0.287	0.0368	7.79	<0.001	0.708
Inspection actions have allowed for improved attention and counseling to families.	0.874	0.890	0.163	0.0356	4.59	<0.001	0.497
Supervision of the use of ICT by teachers.	0.876	0.894	0.404	0.0504	8.02	<0.001	0.693
Supervision of the use of ICT by guidance counselors.	0.876	0.894	0.505	0.0575	8.78	<0.001	0.810
Supervision of the use of ICT by educational guidance teams.	0.878	0.894	0.480	0.0618	7.77	<0.001	0.737
Attention and inclusion of diversity (AID)							
Inspection actions allow for improved transmission of relevant information to families.	0.875	0.891	0.194	0.0330	5.89	<0.001	0.562
Inspection actions allow for improved management of coexistence.	0.877	0.893	0.188	0.0300	6.29	<0.001	0.592
Inspection actions allow for improved reductions in dropout rates.	0.875	0.892	0.346	0.0451	7.67	<0.001	0.707
Inspection actions allow for improved individualized and adapted attention to students.	0.877	0.892	0.232	0.0293	7.92	<0.001	0.702
Review of programming and methodology in schools after each evaluation.	0.880	0.896	0.139	0.0449	3.09	0.002	0.311
Control of school absenteeism.	0.880	0.895	0.319	0.0517	6.17	<0.001	0.594
Technological resources (TR)							
Use of the Séneca Platform.	0.880	0.897	0.422	0.0497	8.48	<0.001	0.730
Use of the Inspectio Platform.	0.881	0.897	0.219	0.0501	4.38	<0.001	0.421
Use of email.	0.879	0.896	0.518	0.0512	10.12	<0.001	0.837
Use of the phone.	0.876	0.893	0.440	0.0486	9.06	<0.001	0.771
Use of video conferencing	0.878	0.895	0.178	0.0562	3.16	0.002	0.312

Note. SE, standard error; Z, Z-value of the estimation; *p*, *p*-value of the estimation; β, standardized estimation.

**Table 2:** Internal consistency values, means, standard deviations, and correlations (Pearson) for the dimensions of the questionnaire.

Variable	α	ɷ	M (SD)	AMEC	SGTA	AID	TR
AMEC	0.86	0.83	16.84 (±2.71)	-	0.41 **	0.54 **	0.27 **
SGTA	0.83	0.85	10.47 (±2.39)		-	0.36 **	0.32 **
AID	0.73	0.79	10.32 (±1.64)			-	0.18 *
TR	0.75	0.84	11.43 (±2.04)				

Note: (1) M, mean; SD, standard deviation; AMEC, attention to members of the educational community; SGTA, supervision of guidance and tutorial action; AID, attention to and inclusion of diversity; TR, technological resources. (2) * *p* < 0.05; ** *p* < 0.01.

**Table 3:** Mean differences according to gender.

Variables	Men (n = 88) M (SD)	Women (n = 30) M (SD)	*t* (116)	*p*	Effect (*d*)
AMEC	16.84 (±2.86)	16.85 (±2.24)	0.030	0.148	0.003
SGTA	10.34 (±2.56)	10.83 (±1.82)	0.953	0.066	0.220
AID	10.31 (±1.69)	10.35 (±1.53)	0.112	0.223	0.024
TR	11.26 (±2.12)	11.93 (±1.74)	1.353	0.067	0.345

Note: (1) M, mean; SD, standard deviation; AMEC, attention to members of the educational community; SGTA, supervision of guidance and tutorial action; AID, attention to and inclusion of diversity; TR, technological resources. (2) *p* < 0.05. (3) The statistical effect size is expressed as Cohen’s value.

**Table 4:** Mean differences according to age.

Variable	<41 Years M (SD)	41–50 Years M (SD)	51–60 Years M (SD)	>60 Years M (SD)	F	*p*	*η* ^2^	*ω* ^2^
AMEC	16.19 (±2.16)	15.13 (±3.32)	17.18 (±2.54)	17.66 (±2.14)	3.58	0.011 *	0.109	0.095
SGTA	8.60 (±1.48)	9.65 (±2.27)	10.85 (±2.53)	10.91 (±2.06)	6.05	0.015 *	0.089	0.065
AID	8.63 (±1.29)	10.28 (±1.71)	10.51 (±1.54)	10.51 (±1.66)	5.26	0.021 *	0.091	0.066
TR	11.86 (±0.91)	11.19 (±2.18)	11.75 (±2.11)	10.85 (±1.96)	1.72	0.169	0.036	0.010

Note: (1) M, mean; SD, standard deviation; AMEC, attention to members of the educational community; SGTA, supervision of guidance and tutorial action; AID, attention to and inclusion of diversity; TR, technological resources. (2) * *p* < 0.05. (3) The statistical effect size is expressed as Cohen’s value.

**Table 5:** Multilevel regression analysis, criteria variable: attention to and inclusion of diversity.

Criteria Variable	R	R^2^	R^2^ Corrected	F	Predictor Variables	β	*t*
Attention to and inclusion of diversity	0.567	0.322	0.304	27.251			
					AMEC	0.476	5.652 **
					SGTA	0.170	2.019 **

Note: (1) AMEC, attention to members of the educational community; SGTA, supervision of guidance and tutorial action. (2) ** *p* < 0.01.

**Table 6:** Regression weights and standardized regression weights for men.

Relationships between Variables	Estimations	R.W. E.E.	C.R.	*p*	S.R.W. Estimations
AID <--> AMEC	0.415	0.134	3.107	***	0.361
AID <--> SGTA	0.199	0.151	1.320	0.187	0.153
TR <--> AID	0.456	0.114	3.984	***	0.394
AMEC <--> SGTA	0.051	0.110	4.629	***	0.574

Note: (1) R.W., regression weights; S.R.W., standardized regression weights; E.E., error estimate; C.R., critical ratio. (2) AMEC, attention to members of the educational community; SGTA, supervision of guidance and tutorial action; AID, attention to and inclusion of diversity; TR, technological resources. (3) *** *p* < 0.001.

**Table 7:** Regression weights and standardized regression weights for women.

Relationships between Variables	Estimations	R.W.E.E.	C.R.	*p*	S.R.W. Estimations
AID <--> AMEC	0.046	0.195	0.238	0.812	0.044
AID <--> SGTA	0.384	0.190	2.023	***	0.378
TR <--> AID	−0.159	0.247	−0.646	0.518	−0.120
AMEC <--> SGTA	0.027	0.012	2.149	***	0.431

Note: (1) R.W., regression weights; S.R.W., standardized regression weights; E.E., error estimate; C.R., critical ratio. (2) AMEC, attention to members of the educational community; SGTA, supervision of guidance and tutorial action; AID, attention to and inclusion of diversity; TR, technological resources. (3) *** *p* < 0.001.

## Data Availability

Data are available upon justified request to the corresponding author.
